# LAVASET: Latent Variable Stochastic Ensemble of Trees. An ensemble method for correlated datasets with spatial, spectral, and temporal dependencies

**DOI:** 10.1093/bioinformatics/btae101

**Published:** 2024-03-04

**Authors:** Melpomeni Kasapi, Kexin Xu, Timothy M.D. Ebbels, Declan P. O’Regan, James S. Ware, Joram M. Posma

**Affiliations:** 1 Section of Bioinformatics, Division of Systems Medicine, Department of Metabolism, Digestion, and Reproduction, Faculty of Medicine, Imperial College London, London W12 0NN , United Kingdom; 2 Faculty of Medicine, National Heart & Lung Institute, Imperial College London, London W12 0NN, United Kingdom; 3 MRC London Institute of Medical Sciences, Imperial College London, London W12 0HS, United Kingdom; 4 Royal Brompton & Harefield Hospitals, Guy’s and St. Thomas’ NHS Foundation Trust, London SW3 6NP, United Kingdom; 5 Program in Medical & Population Genetics, Broad Institute of MIT & Harvard, Cambridge, MA 02142, United States

## Abstract

**Motivation:**

Random forests (RFs) can deal with a large number of variables, achieve reasonable prediction scores, and yield highly interpretable feature importance values. As such, RFs are appropriate models for feature selection and further dimension reduction. However, RFs are often not appropriate for correlated datasets due to their mode of selecting individual features for splitting. Addressing correlation relationships in high-dimensional datasets is imperative for reducing the number of variables that are assigned high importance, hence making the dimension reduction most efficient. Here, we propose the LAtent VAriable Stochastic Ensemble of Trees (LAVASET) method that derives latent variables based on the distance characteristics of each feature and aims to incorporate the correlation factor in the splitting step.

**Results:**

Without compromising on performance in the majority of examples, LAVASET outperforms RF by accurately determining feature importance across all correlated variables and ensuring proper distribution of importance values. LAVASET yields mostly non-inferior prediction accuracies to traditional RFs when tested in simulated and real 1D datasets, as well as more complex and high-dimensional 3D datatypes. Unlike traditional RFs, LAVASET is unaffected by single ‘important’ noisy features (false positives), as it considers the local neighbourhood. LAVASET, therefore, highlights neighbourhoods of features, reflecting real signals that collectively impact the model’s predictive ability.

**Availability and implementation:**

LAVASET is freely available as a standalone package from https://github.com/melkasapi/LAVASET.

## 1 Introduction

Random forest (RF) classifiers are frequently used to analyse biological data for prediction and feature selection tasks. RFs can deal with a large number of variables, achieve reasonable prediction scores, and yield highly interpretable feature importance values (Breiman 2001). As such, they are appropriate models for feature selection and further dimension reduction (DR) for integrated datasets. The premise of the original RF algorithm is to assemble an ensemble of trees that complement each other and increase variability of predictor selection (Nguyen *et al*. 2021). However, each node and sub-sequent split still only consider one predictor variable, limiting both the predictive ability and correct feature importance assignment in complex biological settings that include correlated features.

Nguyen *et al*. (2021) have recently developed a new information criterion statistic to evaluate the contribution of features to the predictive ability of the model. It comprises different categories of probabilities that assess the feature’s proximity to the target class and the complexity of the relationship with the given class. In addition, permutation-based feature importance has been extensively studied in RFs. It has been demonstrated that there is some level of bias in the assignment of feature importance when there exists collinearity between features that are both associated with the target outcome (Nicodemus *et al*. 2010).

Few techniques have been proposed for enhancing feature importance calculations in datasets with highly correlated variables. The Boruta algorithm (Kursa and Rudnicki 2010) uses a ‘shadow’ feature approach where it duplicates the original dataset and shuffles the feature values, however, permuting the values it does not take into account local correlations. Boruta trains a classifier on the enhanced dataset and assigns a value of importance to each of the features, those with lower importance than their permuted counterparts are removed before repeated the process with a new random state. Boruta, in reality, combines permutation importance, by shuffling the original features, with recursive feature elimination (Guyon *et al*. 2002), by iteratively considering and removing features that do not reach a threshold, but its classifier still considers only individual features and the permutation procedure does not consider local correlations.

These methods are efficient in removing noisy features that might not reflect real signals, especially by eliminating these through iterations. However, they are not sensitive in picking all relevant features when these are collinear. Addressing relationships between collinear features in high-dimensional datasets is imperative for reducing the number of features that are assigned high importance and thereby making the DR more efficient. Here, we propose a novel method termed LAtent VAriable Stochastic Ensemble of Trees (LAVASET) that derives latent variables based on the distance characteristics of each feature and thereby incorporates the correlation factor in the splitting step. Hence, it inherently groups correlated features and ensures similar importance assignment for these. Distance characteristics for the features can include the feature’s adjacent points in a 1D spectrum, adjacent features of time-series data, or spatial distance in 3D structures among other examples. In this context, LAVASET addresses a major limitation in the interpretation of feature importance of RFs when the data are collinear, such as is the case for spectroscopic and imaging data.

## 2 Materials and methods

### 2.1 Datasets

We demonstrate the LAVASET algorithm (detailed below) on four different datasets with feature importances calculated as a result of a prediction/classification problem between disease and healthy control (HC) groups or simulated groups.

#### 2.1.1 Irritable bowel syndrome—faecal metabolomics (1D)

The Maastricht University Irritable Bowel Syndrome (MIBS) cohort includes human faecal water samples analysed with ^1^H Nuclear Magnetic Resonance (NMR) spectroscopy for 267 participants (146 IBS patients; 121 HCs). Details on demographics, sample collection, and data acquisition can be found in Mujagic *et al*. (2022)). Unlike the original publication, we used the full NMR spectrum (digitized to a total of 18 600 features) following the removal of the internal standard and the water region, and baseline correction.

To test LAVASET’s ability in capturing relevant features, we also created simulated groups from the MIBS cohort dataset. The groups were generated by using two (uncorrelated) compounds that each have two multiplets, with signals spread out across the length of the spectrum. The compounds used are metabolites ethanol, with peaks at 1.17–1.20 and 3.64–3.68 ppm, and uracil with peaks at 5.79–5.81 and 7.53–7.56 ppm.

#### 2.1.2 Diabetes—urinary metabolomics (1D)

Human urinary metabolomics data from individuals with type-2 diabetes mellitus (T2DM), freely available from Metabolights (MTBLS1), were used as an additional test cohort. Prior work on this dataset has shown higher classification accuracy compared to IBS data. A total of 84 samples were collected, consisting of 12 healthy volunteers with data at seven time points, and 30 individuals with T2DM with data collected at 1–3 time points (total of 50 spectra). These were analysed by ^1^H-NMR spectroscopy to evaluate the urine profiles between T2DM and HCs. Metabolite identification was performed by PLS-DA models previously, as described by the authors in Salek *et al*. (2007)). The raw data were downloaded from MTBLS1 and processed to standardize each spectrum to 18 000 data points. The water and internal standard regions were removed from the spectrum with the remaining points used for the modelling.

#### 2.1.3 Acute myocardial infarction—electrocardiogram (1D)

Electrocardiogram (ECG) data from the Physikalisch-Technische Bundesanstalt (PTB) dataset (Bousseljot *et al*. 2009) were downloaded from PhysioNet (Goldberger *et al*. 2000). We extracted ECG data from individuals with acute myocardial infarction (MI) and compared this against no acute MI. We excluded all data without a reason for admission or with an unknown diagnosis. This resulted in 175 control and 346 acute MI ECGs. The individual ECGs were processed to correct signal drifts. An average cardiac cycle was extracted for each individual that was normalized to 750 data points (0.75 s). We used the three Frank leads [vector cardiogram (VCG)] as input to the algorithm and visualize the feature importance in the conventional 12-leads by making use of the (absolute) Kors regression transformation (Kors *et al*. 1990) for the eight independent leads.

#### 2.1.4 Hypertrophic cardiomyopathy—CMR imaging (3D)

To test LAVASET’s performance on high dimensional, spatial, 3D datasets, we used data meshes derived from cardiac magnetic resonance (CMR) imaging of the human left ventricle. Segmentation of these images (to produce the meshes) was performed using a deep-learning framework developed by collaborators (Bai *et al*. 2018). Measurements of myocardial wall thickness were calculated along radial segments that connected the inner endocardial and outer epicardial surfaces (Duan *et al*. 2019). This process produced a 46 808 × 4 matrix, where for each of the 46 808 points there is a value for the *x, y*, and *z* coordinates of the ventricle, and the wall thickness at that point. These segmentations were performed on images in the UK Biobank dataset (Sudlow *et al*. 2015) and an in-house Hypertrophic Cardiomyopathy (HCM) cohort [Royal Brompton Hospital Cardiovascular Biobank (Curran *et al*. 2023)]. The total number of samples used here was 1273, consisting of 634 HCM patients and 639 demographically-matched HCs.

### 2.2 Approach

**Figure 1:**
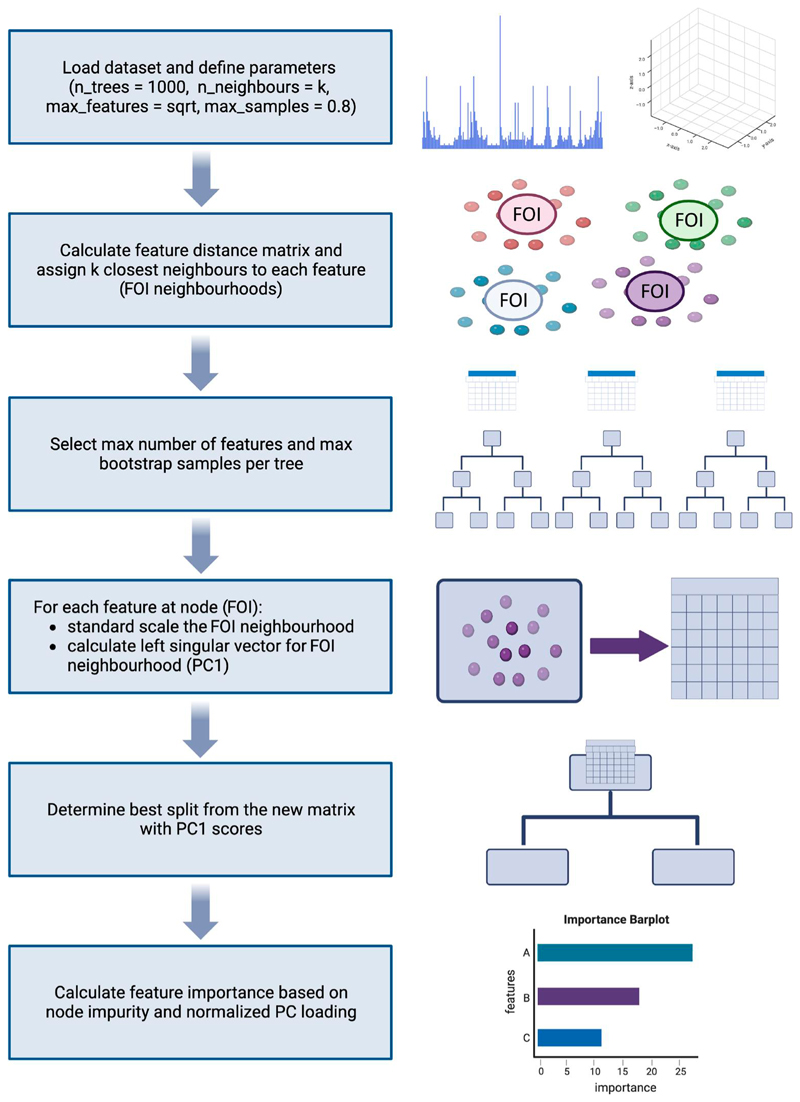
LAVASET high-level pipeline indicating the different and novel approach in the node splitting step and feature importance calculation. Model input is customizable and LAVASET can perform on different types of omics data, from 1D to 3D.

LAVASET operates given a number of prerequisites and hyperparameters that can be optimized (Fig. 1). A user-specified distance matrix is calculated to select the *k* closest feature points to the feature of interest (FOI), which form the FOI neighbourhood. This FOI neighbourhood sub-matrix is defined as *FOIn* = *M*_:, 𝒩 (1,*k*_) where *M* denotes the matrix containing all the input features and the set 𝒩 (1, *k*) represents the indices of the *k* features in *M* that are closest to FOI (based on the user-specified distance matrix), including the index for FOI itself. The user specifies the maximum number of features to consider for each split (with default the square root of the total number of features), and these are randomly selected from the entire dataset in each step. For each selected FOI, the first left singular vector (PC1) of the respective FOI neighbourhood is calculated. We calculate this via Singular Value Decomposition of the sub-matrix *FOIn* = *U*Σ*V*^*T*^. Here, FOIn is the scaled FOI neighbourhood for the selected VOI, *U* is the matrix of left singular vectors, Σ is a diagonal matrix containing singular values, and *V*^*T*^ (or *V* transposed) is the matrix of right singular vectors. The PC1 or first left singular vector is the first vector in matrix *U*, and this is now the latent variable for the FOIn. Loadings for each latent variable are calculated as *L = U*_1_ . *FOIn*, where the first left singular vector *U*_1_ is multiplied by the original FOIn sub-matrix. The input matrix for determining the best split will now consist of the latent variable values instead of the original feature values. The best-split variable and value are evaluated by the traditional Gini index method by deducting the sum of the squared probabilities of each class from one. Once the split occurs, the Gini gain is calculated for the selected latent variable and node by subtracting the sum of the Gini index weights of the two child nodes from the parent node. This is repeated recursively until all samples are split into pure leaf nodes, similar to the classic CART algorithm (Breiman *et al*. 1984).

### 2.3 Feature importance calculation

Feature importance scores are calculated for each feature by weighing its contribution to the PC score (left singular vector). Specifically, for every selected feature where the PC score is calculated, the loadings vector *L* of the score (with a shape equalling the number of neighbours considered) is calculated and multiplied by the Gini gain value assigned to the selected latent variable. This results in a feature importance score not only for the FOI but also for its neighbours.

The LAVASET algorithm is designed to allow for multiple variations in the calculation of a feature’s importance after model construction. LAVASET outputs for each feature_*i*_ a vector of values consisting of three parts. $\Sigma_{t=1}^{T}\;evaluated_{t}$ represents the summation over all trees from 1 to *T* of the count of times a feature is evaluated in each tree and, similarly, $\Sigma_{t=1}^{T}\;selected_{t}$ and $\Sigma_{t=1}^{T}\;gini_{t}$ represent the total counts of times a feature is selected for a split and the accumulated Gini values in each tree, respectively. All features of the original matrix *M* are equally ‘evaluated’ for a split with a frequency that follows a Gaussian distribution. The subset of features ‘selected’ for splitting a node are those assigned a feature importance.

From the feature importance values, we can calculate a normalized Gini feature importance by dividing by the sum of all importances. We also evaluate the ratio of the number of times a feature is selected over the count of times it is considered for a split, and incorporate this in our feature importance final value. Using the same output, another use case would be to compare the ratio of Gini values over the count of times a feature is selected for split, which can give an idea of the value magnitude assigned to a feature at a given split. Results presented in this article utilise the normalised feature importance multiplied by the ratio of $\Sigma_{t=1}^{T}\;selected_{t}$ over the $\Sigma_{t=1}^{T}\;evaluated_{t}$ for each feature. This is shown below, where importance(*i*) is the importance of the feature *i*, T is the total number of trees, importance(*i*, t) represents the Gini importance of the feature *i* in the tree *t*, and *F* is the total number of features. The sum in the numerator of the first fraction goes over all the trees from 1 to *T* for the feature *i*. The sum in the denominator of the first fraction goes over all the trees and all the features (*f* represents each feature in the feature set), which normalizes the Gini of the feature *i*. The ratio of the sums in the second fraction measures the frequency with which feature *i* was selected when it was evaluated: $importance(i)=\frac{\Sigma_{t=1}^{T}\;gini(i,t)}{\Sigma_{f=1}^{F}\Sigma_{t=1}^{T}\;gini(f,t)}\times \frac{\Sigma_{t=1}^{T}\;selected(i,t)}{\Sigma_{t=1}^{T}\;evaluated(i,t)}$.

### 2.4 Model evaluation

Standard metrics (accuracy, precision, recall, and *F*1-score) are used to compare the classification performance of RFs and LAVASET. For both the simulated and MIBS cohort inputs, LAVASET and RF were run 20 distinct times (20 run pairs with 100 and 1000 trees, respectively). Identical random state seeds were assigned per run pair for the sample bootstrapping, to account for the randomness between the comparisons. The data were split into training and test sets (80% and 20%, respectively), with the sets always being kept identical across the runs. Mean accuracy, precision, recall, and *F*1-scores are reported for the 20 runs.

We performed a grid search optimizing the number of trees and neighbours for the MIBS cohort. Tree values ranged from 100 to 10 000 and neighbours from 1 to 50, and all possible combinations were evaluated. The optimal value for the number of trees for LAVASET was 1000, this model is referred to as LAVASET-1K. We compare LAVASET-1K to two RF models: the first is an RF with the same number of 1000 trees, referred to RF-1K hereafter, and the second is an RF that runs for the same amount of computational time as LAVASET. We evaluated the number of RF trees that can be fit in the same amount of time as LAVASET-1K required. LAVASET-1K fits 1000 trees for 18 neighbours (optimal for MIBS cohort) in ~11 min [on an HP Z6 G4 workstation with 16-core Intel(R) Xeon(R) Silver 4110 CPU @ 2.10 GHz with 128 GB RAM], a classic RF can include ~41 000 trees in the same amount of time (referred to as the RF-41K model).

Precision and recall scores are also calculated as metrics for evaluating the peak coverage of the feature importance performance. In the simulated dataset, the peak points used to create the two distinct groups are assigned as the ground truth (positive designation) for the peak coverage. To evaluate the specificity of LAVASET, we include points outside the peak (equal to *k* neighbours assigned) in the calculations of precision and recall. These serve as the true negative designations. The threshold for positive or negative designation in these calculations is whether the point has been assigned a feature importance value (>0) or not important zero.

The neighbours for the VCG data were calculated on the basis of the time of the cardiac cycle. That is, selecting Frank’s lead *x* for variable (time) *i* also includes the other two leads (*y, z*) at time *i*. Including more neighbours takes place in steps of six (*x, y*, and *z* for both *i ±* 1). Where the first and last time points are also considered neighbours at *i ±* 1.

Finally, we assessed LAVASET’s performance on feature extraction for DR of the 3D CMR dataset. Due to computational constraints, the 3D dataset was tested on 100 and 200 neighbours and 150K trees. Neighbours were decided by the spatial distance (*x, y*, and *z* coordinates) in an iterative manner by considering the 100 closest points that remain neighbours for over half of the 1273 samples. The most important features (above the 50% importance value threshold) were selected for LAVASET and RF models (both 150K trees). For each set, we performed further DR and clustering to evaluate how the clusters separated HCM and HC samples. Uniform manifold approximation and projection (UMAP) and *k*-means clustering with *k* = 2 (expected number of groups) were used in both cases (McInnes *et al*. 2018). UMAP components and *k*-means transformations were evaluated for 20 different random state restarts to ensure robustness. Each restart produced two clusters that were scored by the standard metrics, to determine how closely they matched the true sample labels.

## 3 Results

### 3.1 Simulated NMR dataset

LAVASET yields a non-inferior prediction accuracy compared with traditional RFs when predicting the two simulated groups in the NMR dataset (accuracy: 0.859±0.027 LAVASET, 0.823±0.021 RF, Supplementary Fig. S1). As shown in Supplementary Fig. S2, both the Boruta and RF methods [Panels (B), (C), (E), and (F)] fail to identify all individual features relating to the same peak(s) and instead only identify a subset of these. In contrast, LAVASET not only does identify correctly the simulated metabolites, but also assigns appropriate importance values to all or most of the correlated points that encompass a peak. Specifically, for ethanol’s CH_2_ peak shown in Supplementary Fig. S2A, *F*1-scores for LAVASET are 96% and 85% for the CH_3_ peak. For uracil, the second multi-peak metabolite used to create the simulated dataset (Supplementary Fig. S2D), LAVASET has *F*1-scores at 96% and 89% in the two doublets. In all the metabolite peaks considered here, Boruta and RF underperform in capturing all the points that comprise the peaks (Boruta *F*1-scores: 23%, 52%, 47%, and 60% for each peak, respectively; RF: 31%, 53%, 49%, and 63%).

### 3.2 MIBS cohort

After a grid search evaluation to identify the optimal parameters for the number of neighbours and trees, the highest scoring accuracy was achieved by 1000 trees and 18 neighbours (Supplementary Fig. S3). Average accuracy values overall dropped after considering more than 20 neighbours and there were no significant differences when taking more than 1000 trees for the specific dataset. Supplementary Figure S4A presents the optimal number of neighbours for the 1000 trees with a clear peak being displayed on the graph, while the drop after 20 neighbours is also evident there. LAVASET yields similar results when classifying IBS versus HC in the MIBS cohort (only for this cohort, losing 1% in mean accuracy in comparison to RF). Across 20 runs the average accuracy score for LAVASET is 0.68±0.02, for RF-1K 0.69±0.01, and for RF-41K 0.68±0.02. Supplementary Figure S4B boxplots represent these accuracy values, along with precision, recall, and *F*1 score values. The value ranges shown for the RF-41K model suggest that when running 41 000 trees there is potential for over-fitting, given that the values across each of the 20 runs are almost identical. For the four different performance metrics, the error bars of LAVASET versus RF-1K and LAVASET versus RF-41K over-lap, indicating LAVASET is non-inferior to either RF model (Supplementary Fig. S4B).

**Figure 2:**
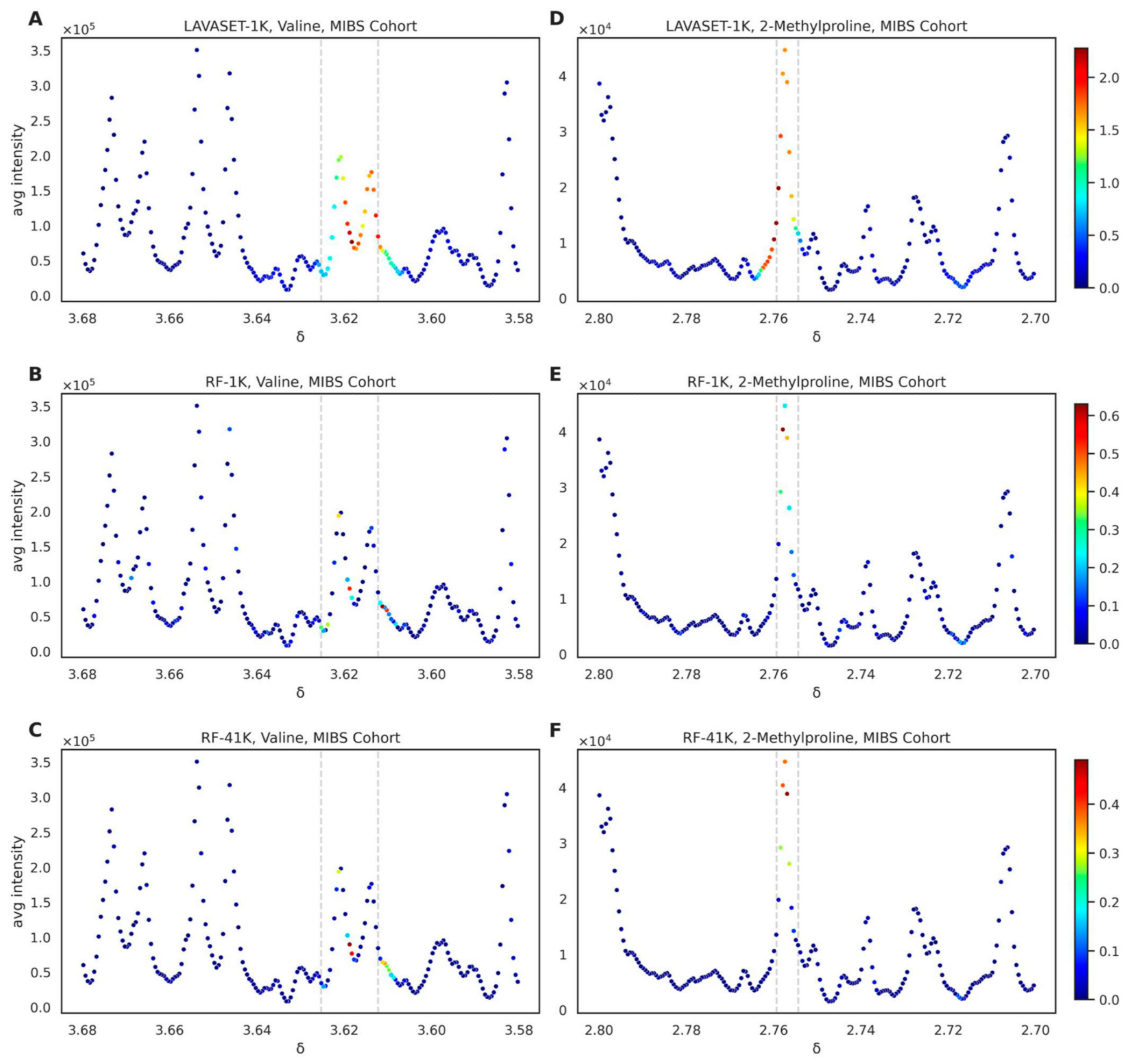
Comparison of LAVASET-1K to RF-1K and RF-41K feature importance assignments on pre-identified metabolites. Panels (A)–(C) show feature importances [as defined in Section 2] for valine, and panels (D)–(F) for 2-methylproline. Colourbar on the right indicates the feature importance value range (red = higher, blue = lower). Dashed grey lines indicate the previously identified points for the specific metabolites peaks. *X*-axis indicates the chemical shift (in parts per million, *δ*). *Y*-axis shows the average signal intensity.

The most pronounced differences between LAVASET and RF are evident when looking at feature importances. LAVASET’s ability to capture the entirety of peaks attributed to a metabolite surpasses the RF feature evaluation which merely captures less than half the points that encompass the peak. Figure 2 shows the relevant peaks of previously identified metabolites valine (A, B, and C) and 2-methylproline (D, E, and F). These two metabolites have been previously associated with separating IBS from HC patients, by showing high feature importance in classification models using Support Vector Classifiers (Mujagic *et al*. 2022). Panels (A) and (D) show clearly how LAVASET is able to assign importance values to all points of the valine and 2-methylproline peaks, respectively. The dashed grey lines indicate the previously identified point ranges for the specific metabolites peaks, which present a ground truth for the peak, but can sometimes be affected by small shifts or missing points on the sides of a given peak. In the case of 2-methylproline, we notice that LAVASET is also capturing and assigning relatively high importance on points to the left side of the peak. These points, however, when visualized on the spectrum appear to be part of the rest of the peak and LAVASET only assigns an importance to the points up to where the next peak is starting, without including that next non-related peak. This can also be seen on the right-side of valine in Fig. 2A, where it shows a gradual decline in importance as we are reaching the end of the visualized peak.

RF, on the other hand, assigns feature importance to sporadic points of the peak, with no real continuance as to the values of importance (valine, Fig. 2B, red point indicating high importance next to light blue point indicating more than half of an importance value). Even in the case of the 41K trees, we see that the points selected remain almost the same as in the 1K trees, suggesting that an RF cannot reach LAVASET’s ability in capturing whole peaks, even if the number of trees increases 41-fold. Overall, LAVASET’s output is quite stable and not affected significantly by small changes in the number of neighbours. Peak coverage is equivalent when looking at 10–16 neighbours, with the main differences occurring in the value of importance to each point (Supplementary Fig. S5).

### 3.3 MTBLS1 cohort

LAVASET was further tested on the MTBLS1 T2DM cohort, as described in Section 2, to ensure that performance stability and feature importance interpretation remain effective in other cohorts. Consistent with previous examples, LAVASET presents non-inferior results to RF (LAVASET accuracy: 0.82±0.01, RF accuracy: 0.77±0.02, Supplementary Fig. S6) and manages to capture the metabolites pre-identified by Salek *et al*. (2007)). Metabolite peak capturing by LAVASET is again superior to RF, by encompassing if not all, most points and attributing feature importance values more equally. Results are expanded in Supplementary Fig. S7).

### 3.4 ECG data

**Figure 3:**
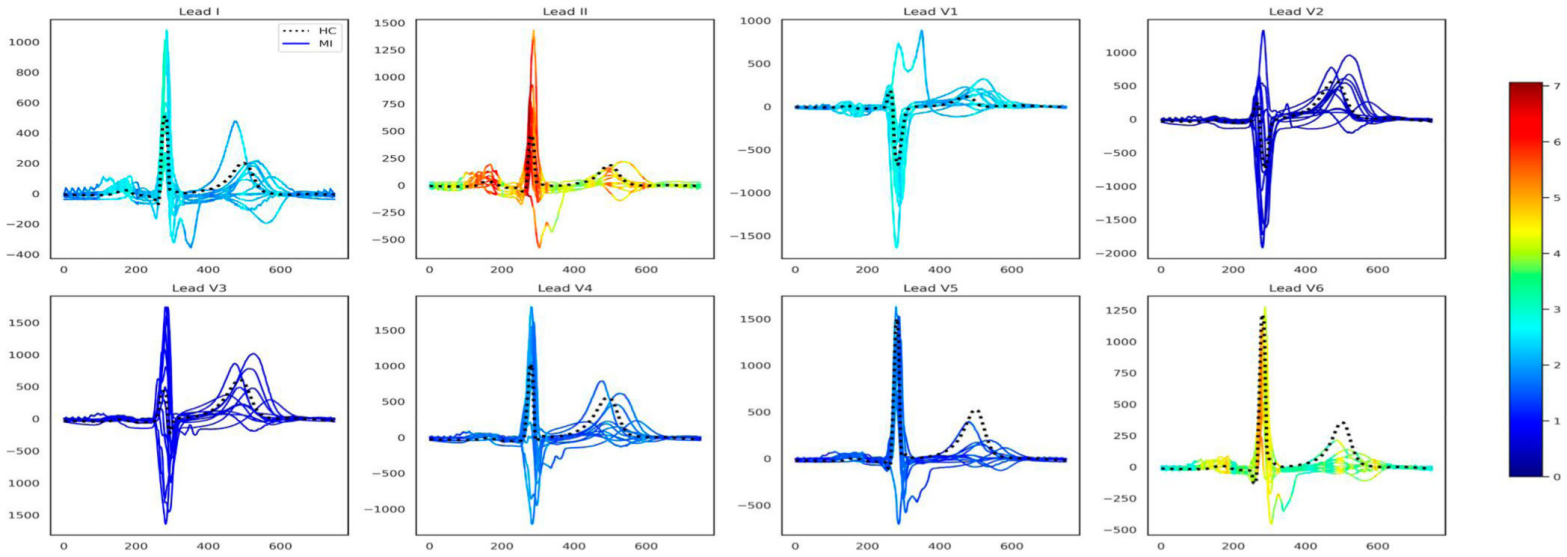
ECG feature importance (Kors regression back-transformed from VCG feature importance) normalized across the eight independent ECG leads. Subplot titles indicate the respective leads. *X*-axes show time in milliseconds and *y*-axes voltage magnitude in millivolts. The dotted black line indicates a healthy sample and the solid lines represent the 10 MI types (acute, anterior, anteriolateral, anteroseptolateral, anteroseptal, inferior, inferolateral, inferoposterolateral, lateral, and posterior) in this dataset and are coloured by relative feature importance.

We assessed LAVASET’s capabilities on more complex data types where it is common practice to use transformations of the raw reading data to infer further information. We transformed ECG readings to VCG inputs. For this task, we used ECG data from the PTB dataset (Bousseljot *et al*. 2009), as described in Section 2. LAVASET-1K performed similarly to the RF-1K in this dataset, with accuracy values showing identical results across 20 runs (0.81±0.01, Supplementary Fig. S8 and other metrics having only small differences (Supplementary Fig. S8). Figure 3 shows the results when taking 20 nearest neighbours (across the time axis as described in Section 2) and 1000 trees. LAVASET is able to capture peaks and anomalies across the ECGs that indicate differences between the HC and MI cases. Given the nature of the dataset and the idiosyncrasies of different MI cases based on the location of the infraction, setting a binary classification by incorporating all types of MI as one target will only capture important features related to all these different MI phenotypes.

### 3.5 CMR imaging

**Figure 4:**
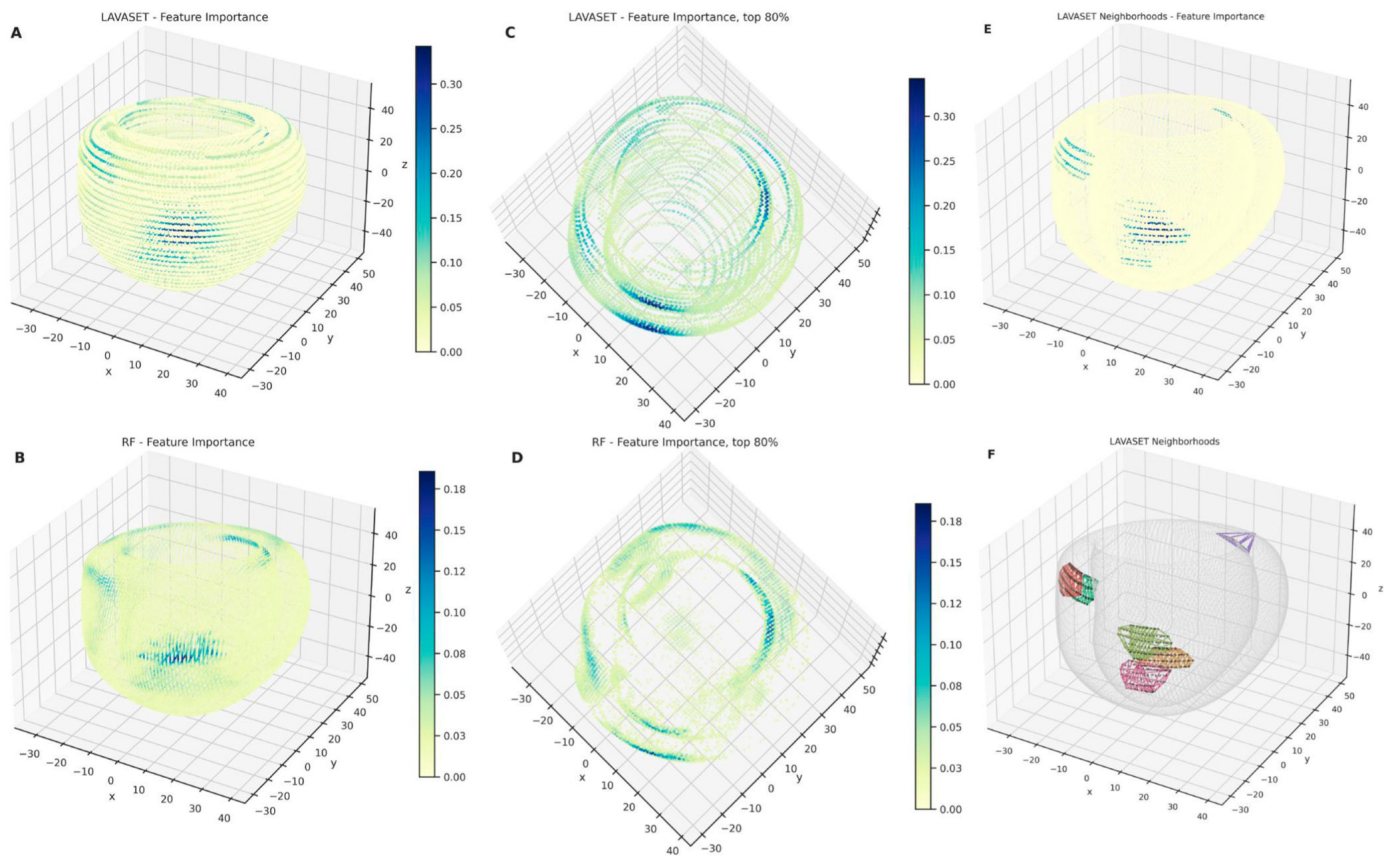
The 3D representations of the left ventricle CMR data points (*x, y*, and *z* coordinates). Points represent an averaged template of HCM and HC ventricles. The inner and outer structures formed show the endocardium and epicardium, respectively. Colourbar shows the feature importance gradient, indicating that in panel (A) (LAVASET) the assignment of higher importance is encompassing the entirety of the ventricle structure. In panel (B), assignments are given in a patch-like manner for RF. Panels (C) and (D) show the points in the 80% quantile from a top view to facilitate the distinction between the inner and outer walls of the ventricle. Panels (E) and (F) show six distinct neighbourhoods of FOIs. In panel (D), convex hulls are drawn for each neighbourhood to represent the pattern of points per neighbourhood. Black points indicate the neighbours and the coloured connecting lines emphasize the different neighbourhoods, and the string-like pattern of neighbour points. Panel (C) shows the feature importance for the respective six neighbourhoods.

Both the LAVASET and RF models demonstrate equivalent values across all metrics. LAVASET shows an accuracy of 0.878, precision of 0.837, recall of 0.904, and *F*1-score of 0.869. RF shows an accuracy of 0.875, precision of 0.916, recall of 0.851, and *F*1-score of 0.882. Given these high values, we are confident about the use of both models for feature extraction, as means to DR. Figure 4 shows the results on 100 neighbours and 150 000 trees. LAVASET is able to assign importance values to a larger surface area of the left ventricle, while also demonstrating the most important areas. RF, on the other hand, picks patches of the left ventricle as most important, disregarding other anatomical parts that could be important. To test how informative these selected features are, we used them as input in further DR and clustering via UMAP and *k*-means. Table 1 shows the clustering performance across 20 iterations of UMAP and *k*-means, indicating that the features selected by LAVASET perform constantly better than those selected by RF. We also highlight that in this specific dataset, feature extraction improves the models considerably, given the significantly lower performance observed when taking all features as input.

## 4 Discussion

We have presented a novel ensemble learning method aimed at datasets with spatial, spectral or temporally dependent relationships between features that enhances variable importance and ensures that correlated features are evaluated appropriately and not independently. LAVASET produces non-inferior performance results to traditional RFs in all but one of the examples tested above, and in both simulated and real datasets. While not sacrificing performance in most examples, it is able to assign feature importances in a superior way, by not missing features that are as ‘important’ by means of correlation, and ensuring that importance values are correctly distributed. One of the advantages of RFs in comparison to other classification methods is its ability to assign feature importances to the original features; here, LAVASET retains this advantage by directly assigning importances to the original feature sets, using the loadings derived from singular vector decomposition to guide the relative importances. In contrast, we have shown that RF allocates feature importance to isolated points, whether that is peak points in a spectrum or points on a 3D mesh, without any clear consistency of the magnitude of importance. Even when substantially increasing the number of trees in RF, the selection of points and assignment of importance are nearly identical to those selected with the lower number of trees, and still do not capture the points selected by LAVASET. In this scenario, Boruta (Kursa and Rudnicki 2010) shows results similar to RFs and LAVASET demonstrates improved feature recovery. Other decision forest methods, such as GrandForest (Larsen *et al*. 2020) and DFNet (Pfeifer *et al*. 2022), that use a graph-structure as input for creating each tree still consider only single features at each split. LAVASET combines correlated features in the splitting step and uses the distance matrix to select the groups of features. When specifying subgraphs for datasets with spatially coherent features, the trees of GrandForest and DFNet will get stuck in local neighbourhoods (both with connected subgraphs and random walks). These methods work better with sparser graphs, hence splitting data further using related features do not add much predictive ability as these features are correlated and the residual of the initial split will not contain much information that can be explained by features correlated to the initial one.

The motivation behind developing LAVASET stems from the idea of enhancing traditional RFs, in a manner similar to how Group Lasso enhances the Lasso algorithm. In the main premise of Group Lasso, we also assume that there are groups of features that are expected to have similar effects or are related to each other. By penalizing the sum of the absolute values (L1 norm) of the coefficients within each group, Group Lasso encourages the model to select entire groups of features together or exclude them altogether (Yuan and Lin 2005). Like in Group Lasso, LAVASET is particularly useful when dealing with high-dimensional data where groups of features exhibit similar importance or are structured in some meaningful way. This was evident by the variety of datasets we tested, where the number of features ranged from 750 to 46K. In this high-dimensional context, LAVASET exhibits stability in its output and remains relatively unaffected by minor variations in the number of neighbours comprising the groups. However, Group Lasso requires non-overlapped groups of features, whereas in LAVASET this can be varied. In fact, in LAVASET, different FOIs can have different numbers of neighbours to increase flexibility. Likewise, LAVASET emulates the kernel filter in convolutional neural networks (CNNs) in that it combines multiple features into a single output (for splitting in LAVASET), however, it does so without condensing the output and attributing the feature importance across the initial features. Other work has investigated the relations between individual features in terms of the similarity of performance at different splits. This methodology is able to discern correlations between individual features, however the mutual forest impact is constrained to evaluating pairs of features only (Voges *et al*. 2023). LAVASET can, in theory, be combined with this to perform *a posteriori* analysis of evaluating correlations between groups of latent variables.

LAVASET’s main limitation derives from the requirement of defining the aforementioned ‘groups’ (akin to the kernel size in CNNs). This parameter is user-specified and assumes an understanding of the relationship between the variables. In the examples presented here, this relationship is defined and assigned by distance, whether that is 1D distance across a spectrum, 1D across time-series data, or 3D spatial distance. The influence of neighbourhood definition is evident, especially in the 3D CMR example. Figure 4F shows that the string-like pattern of importances calculated by LAVASET is predominantly driven by the assignment of neighbours for each FOI. This inherent limitation, however, is what enables LAVASET’s flexibility in creating the groups of neighbours. Distance is only one of the metrics that can be employed. Other examples include genomic distance (combine SNPs via linkage disequilibrium), mass spectrometry isotope patterns (proteomics and metabolomics), or hierarchical relationships (i.e. taxonomy of microbiota). This renders LAVASET more versatile than other similar methods, while giving the user the ability to tailor the algorithm to their specific needs. Hence, it is applicable to a wide variety of datasets and biological questions.

This flexibility is also translated to LAVASET’s code implementation. The algorithm also exploits parallelization in order to speed up computations. The body of the code is written in Python 3.10, while utilizing established C++ scripts for efficiency. Given the nature of the code and algorithm, LAVASET can be run in batches, if needed, or can be easily altered to incorporate additional metrics to distance. Moreover, while beyond the scope of this work, there is potential to explore new hybrid methods; e.g. LAVASETs output and the important features can be used to define new regions of latent features which can then be evaluated in terms of their relations with other regions as part of a graph (and used as input to GrandForest and/or DFNet), and also to consider permuted latent features at each split for simultaneous feature selection (as with Boruta). We have not performed hyperparameter optimization in this work (except for determining the optimal number of neighbours) to allow comparing the different methods like-for-like, however, we envision that in future the number of neighbours in LAVASET is considered in a hyperparameter optimization setting alongside the max depth, number of trees, and others.

To enhance and expand LAVASET’s capabilities, we are working on incorporating the gradient boosting algorithm as one of our built-in additional components. This will extend LAVASET’s core methodology to other ensemble methods and benefit from iterative learning and the specific advantages of boosting trees.

## 5 Conclusion

We have presented LAVASET, a novel ensemble method capable to improve feature interpretability by selecting relevant groups of features instead of individual features. Its novel functionality is most useful in datasets with correlations between features. In cases where this is missing from the data, then traditional RFs are more appropriate. LAVASET offers interoperability to the user both by the structure of its code and via the neighbour’s parameter. It can be applied to almost all omics data types to identify all relevant known or unknown important features and effectively perform feature extraction for DR.

## Supplementary Material

supplementary data

## Figures and Tables

**Table 1:** HCM CMR dataset, feature DR performance.^a^

	LAVASET features	RF features	All features
Accuracy	**0.890**±0.01	0.870±0.002	0.797±0.004
Precision	0.905±0.005	**0.913**±0.004	0.817±0.006
Recall	**0.859**±0.009	0.816±0.003	0.763±0.011
*F*1-score	**0.881**±0.004	0.862±0.002	0.789±0.005

a Scores for accuracy, precision, recall, and *F*1-score across 20 iterations of UMAP and *k*-means (*k* = 2) on the selected feature sets by importance in LAVASET, RF, and without selection. Values shown are the mean±standard deviation. Values in bold indicate the highest performances for each metric.
