# Supplementary material for: LAVASET: Latent Variable Stochastic Ensemble of Trees. An ensemble method for correlated datasets with spatial, spectral, and temporal dependencies
Source: Bioinformatics. Author manuscript; Available in PMC 2024 Jun 28. (PMC11212485; doi:10.1093/bioinformatics/btae101)
Supplement: supplementary data [file EMS197090-supplement-supplementary_data.pdf]

LAVASET: Latent Variable Stochastic Ensemble of Trees. An ensemble method for correlated datasets with spatial, spectral, and temporal dependencies.

Melpomeni Kasapi [1,2], Kexin Xu [1], Timothy M.D. Ebbels [1], Declan P. O'Regan [2,3], James S. Ware [2,3,4,5], Joram M. Posma [1]

[1] Section of Bioinformatics, Division of Systems Medicine, Department of Metabolism, Digestion, and Reproduction, Faculty of Medicine, Imperial College London, London, W12 0NN, UK.
[2] National Heart & Lung Institute, Imperial College London, London, W12 0NN, UK.
[3] MRC London Institute of Medical Sciences, Imperial College London, London, W12 0HS, UK.
[4] Royal Brompton & Harefield Hospitals, Guy's and St. Thomas' NHS Foundation Trust, London, SW3 6NP, UK.
[5] Program in Medical & Population Genetics, Broad Institute of MIT & Harvard, Cambridge, MA, US.

1. SIMULATED NMR DATASET

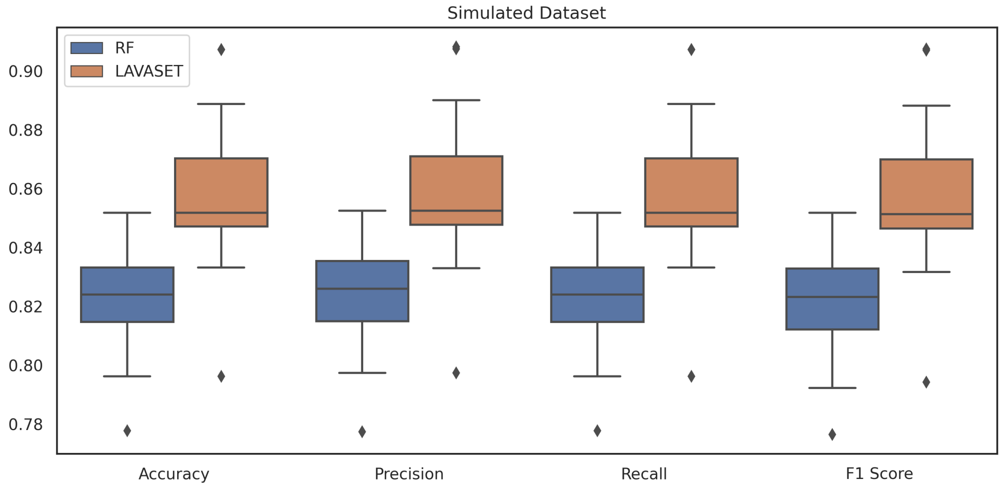

Fig. S1. Performance in the simulated dataset. Models were run for binary classification on simulated groups generated from the MIBS dataset. LAVASET outperforms RF across all metrics as seen in 20 distinct iterations with identical random seeds between LAVASET and RF. Accuracy: LAVASET: 0.859±0.027 , RF: 0.823±0.021. Precision: LAVASET: 0.860±0.026, RF: 0.824±0.020, Recall: LAVASET: 0.859±0.027, RF: 0.823±0.021, F1 Score: LAVASET: 0.859±0.027, RF: 0.823±0.021.

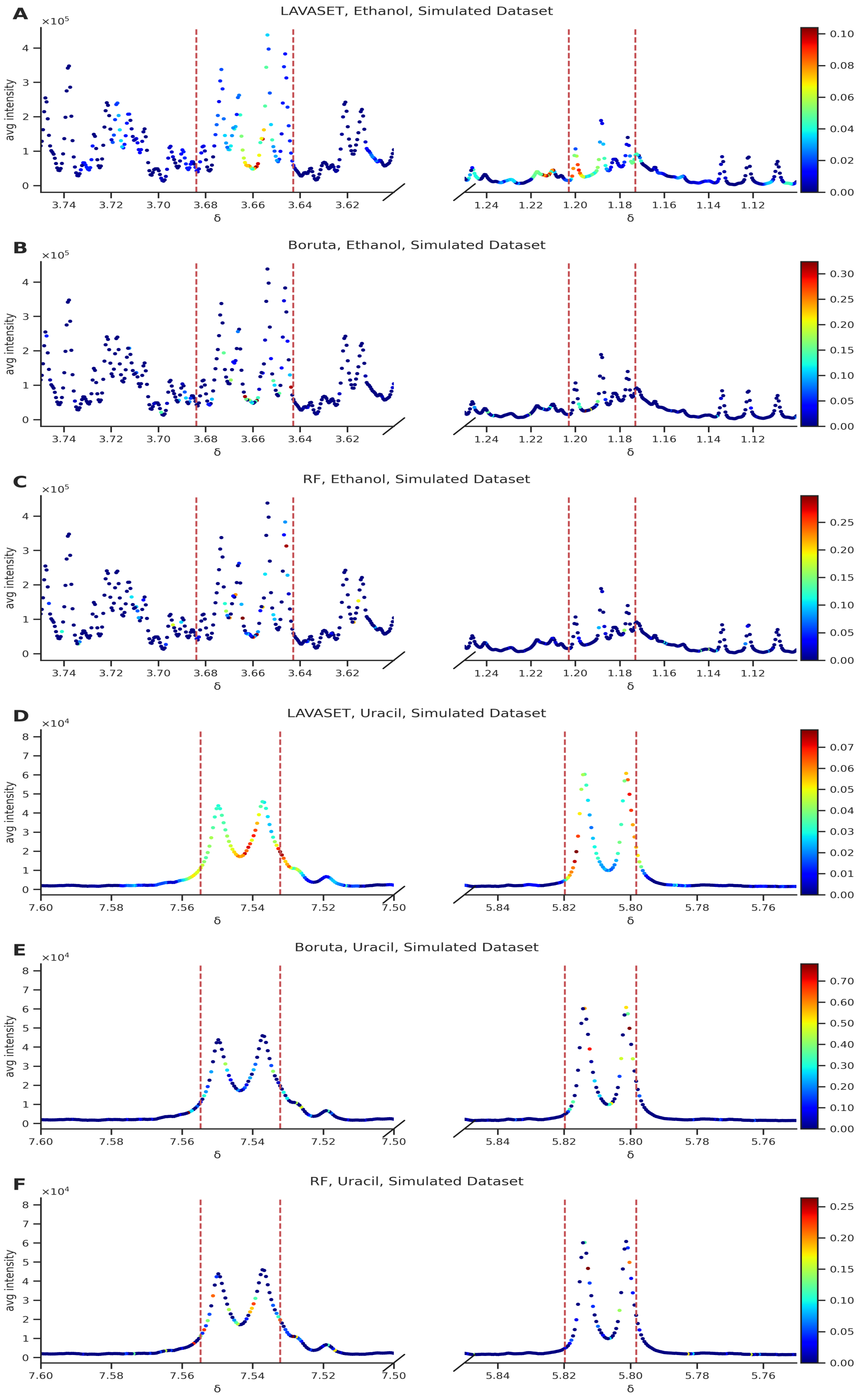

Fig. S2. LAVASET feature importance assignments on the simulated dataset. A close-up look at the specific spectral widths for ethanol and uracil, the top metabolites used to separate two groups in this simulated dataset. LAVASET shows better performance in capturing most points of the peak versus both the Boruta and RF methods which identify less than half. Specifically: Ethanol - LAVASET: CH_2 peak: precision: 0.92, recall: 1.00, CH_3 peak: precision 0.77, recall: 0.95, Boruta: CH_2 peak: precision: 0.86, recall: 0.37, CH_3 peak: precision 0.89, recall: 0.13, RF: CH_2 peak: precision: 1.00, recall: 0.36, CH_3 peak: precision: 1.00, recall: 0.18, Uracil - LAVASET: H-6 (next to C=O) doublet: precision: 0.92, recall: 1.00, H-5 (next to NH) doublet: precision: 0.80, recall: 1.00, Boruta: H-6 (next to C=O) doublet: precision: 0.85, recall: 0.32, H-5 (next to NH) doublet: precision: 0.81, recall: 0.47, RF: H-6 doublet: precision: 1.00, recall: 0.32, H-5 doublet: precision: 0.94, recall: 0.47. Colourmap bar to the right indicates feature importance Gini values, x-axis is marked by the parts per million metric (ppm or δ). The simulated data were run on 100 trees.

2. MIBS COHORT

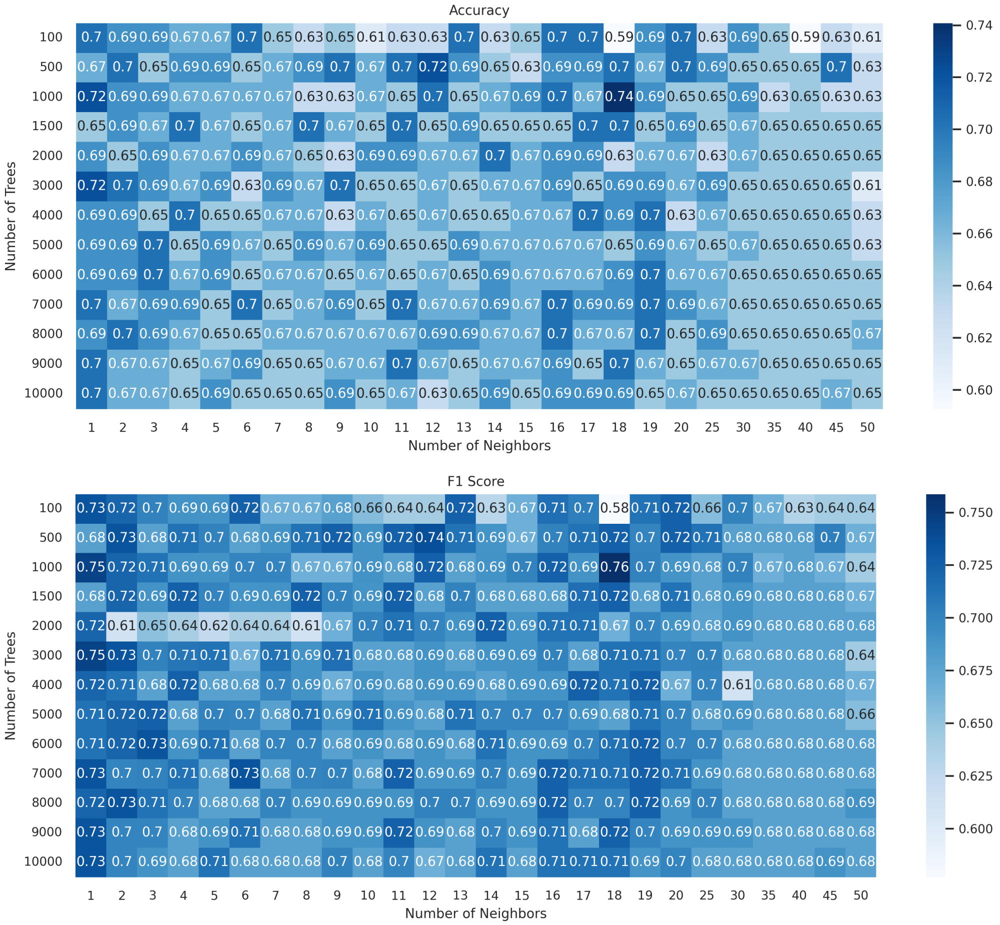

Fig. S3. Accuracy, precision, recall and F1-score values for grid search parameter tuning in MIBS dataset, when performing binary classification of IBS vs HC.

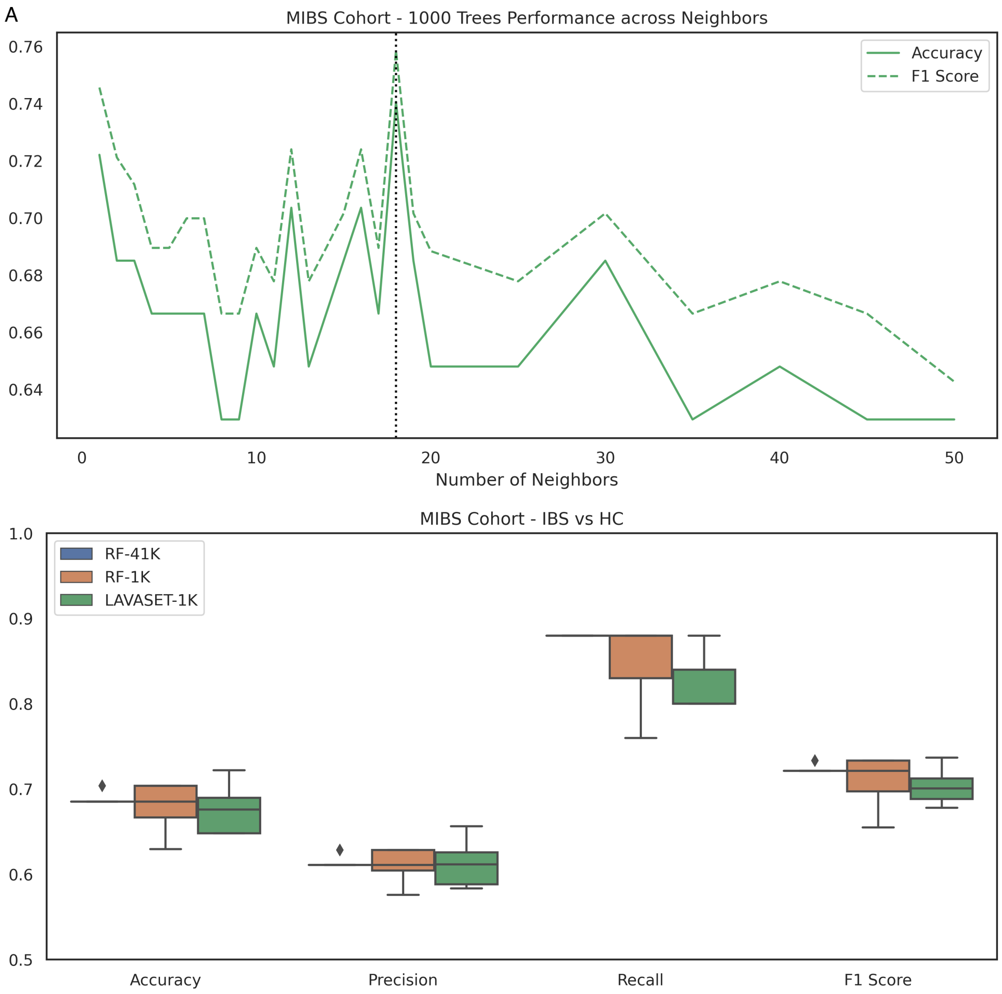

Fig. S4. Panel A shows the changes in performance when looking at a different number of neighbours, evaluated by LAVASET-1K trees, in the MIBS cohort. Solid line presents the accuracy and dashed line presents the f1 score, the black vertical line indicated the best-performing number of neighbours at k=18. Panel B compares the performance of the LAVASET 1,000 trees / 18 neighbours (green) to the RF-1K trees model (blue) and the RF-41K model (time comparison, orange).

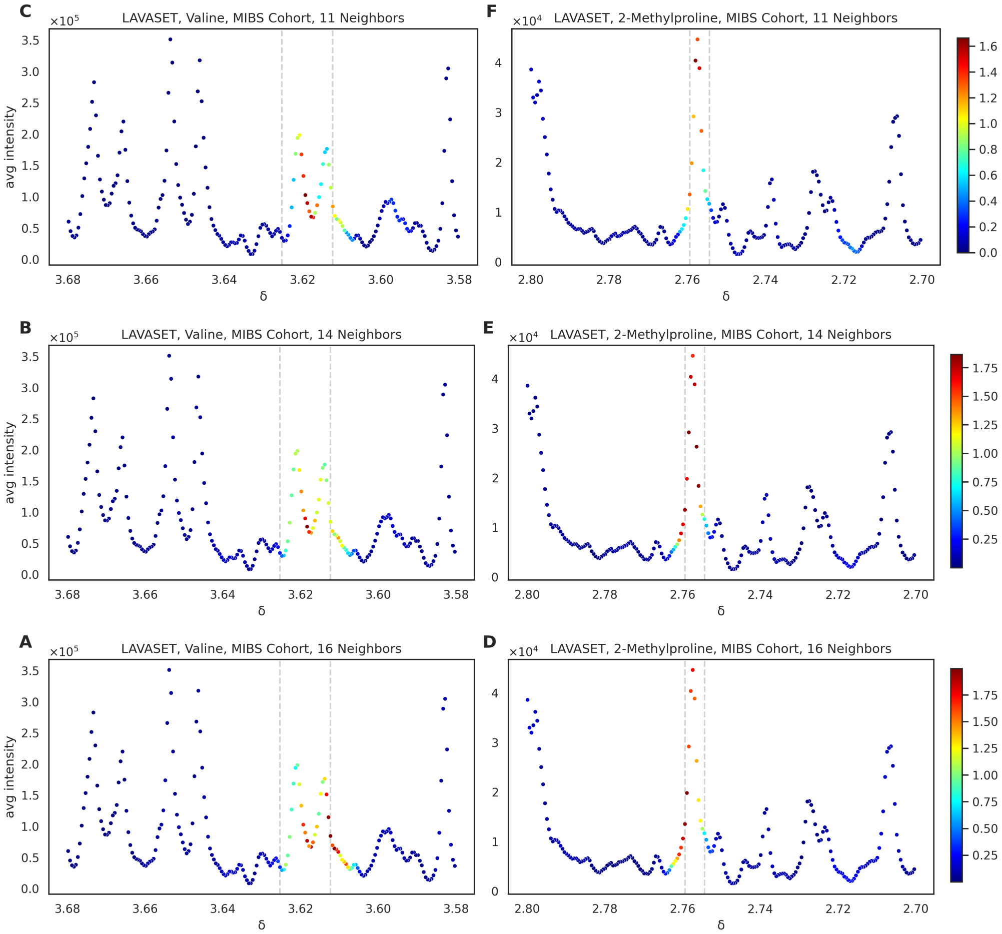

Fig. S5. Feature importance coverage across different number of neighbours for the MIBS cohort.

3. MTBLS1 COHORT

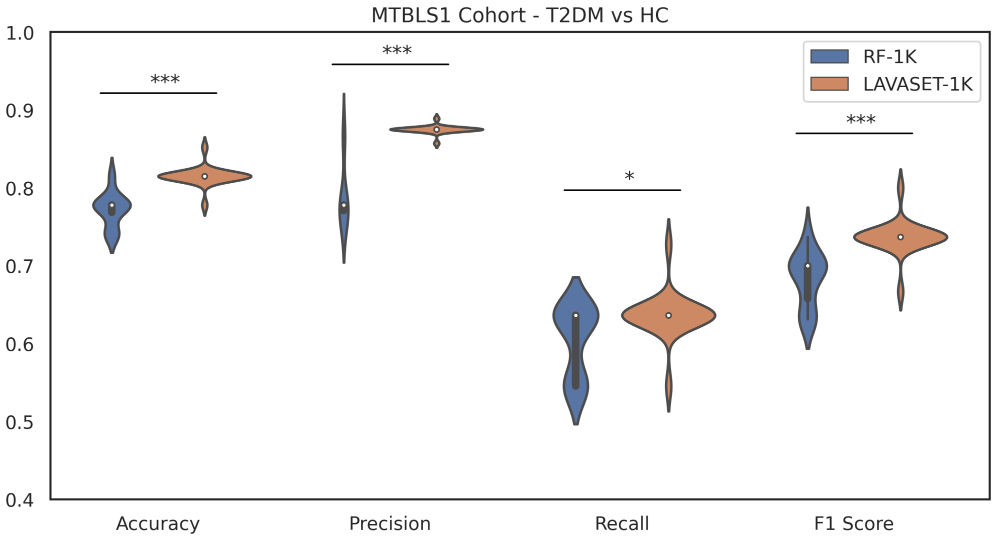

Fig. S6. Performance in the MTBLS1 dataset. Models were run for binary classification between Type 2 Diabetes Mellitus (T2DM) samples and Healthy Controls (HC). LAVASET outperforms RF across all metrics, as seen in 20 distinct iterations with identical random seeds between LAVASET and RF. Accuracy: LAVASET: 0.82±0.01, RF: 0.77±0.02, Precision: LAVASET: 0.88±0.01, RF: 0.79±0.04, Recall: LAVASET: 0.64±0.03, RF: 0.61±0.04, F1-score: LAVASET: 0.74±0.02, RF: 0.68±0.03.

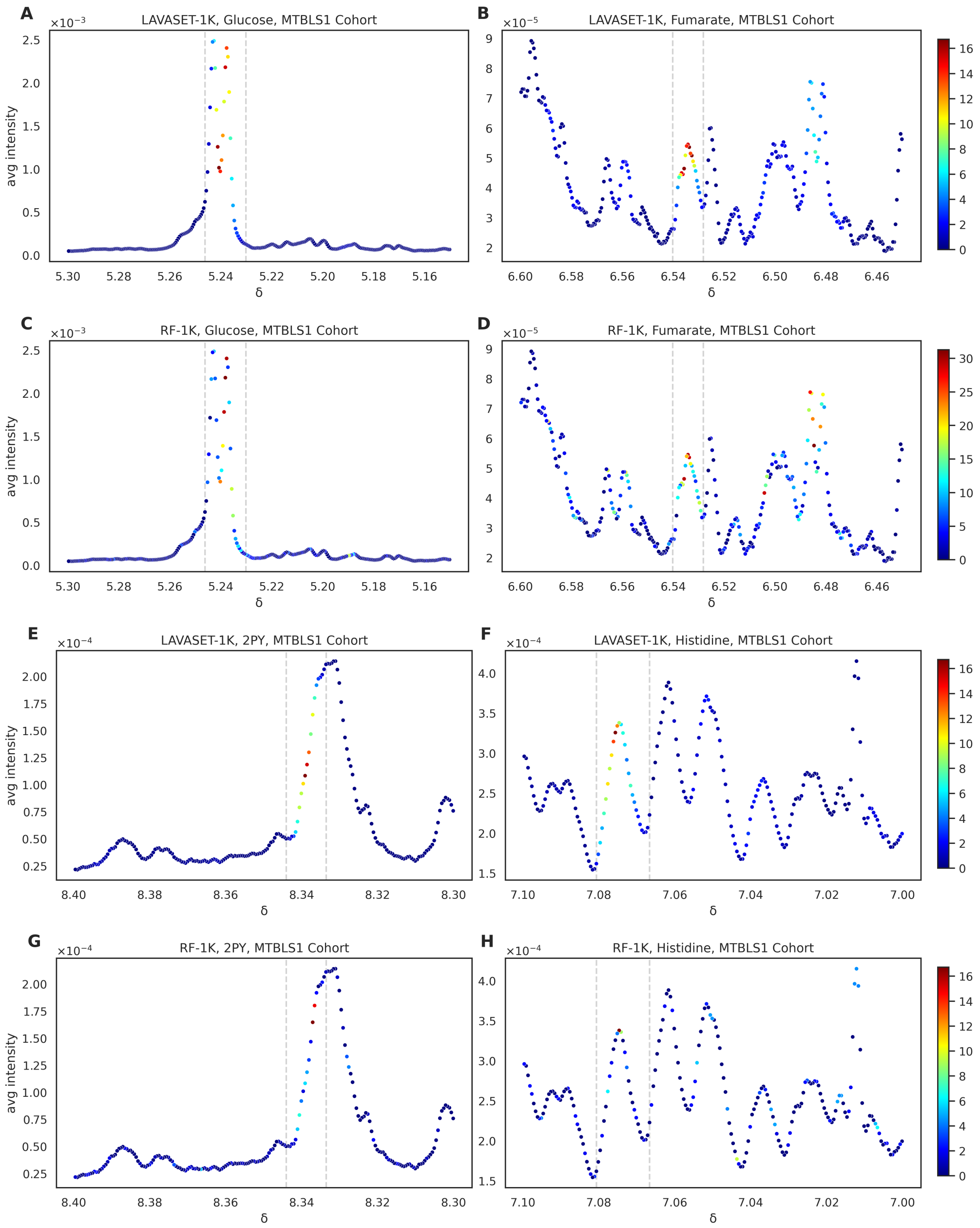

Fig. S7. Feature importance coverage in the Type 2 Diabetes Mellitus (T2DM) MTBLS1 dataset. All figures show results of LAVASET and RF models across 1,000 runs and 10 neighbours (for LAVASET). Panels A and C present glucose coverage in LAVASET and RF respectively, as a peak capturing baseline given the classification was done between T2DM samples and HC. Panels B/D present fumarate, E/G *N*-methyl-2-pyridone-5-carboxamide (2PY) and F/H histidine, all three metabolites are identified in the original cohort study. Gray dashed lines represent the ppm values reported in the study. Additional points around the peak are shown to capture LAVASET's ability of not assigning high importance values on points around the peak (noise). This is evident in the fumarate and histidine examples where RF captures points outside the identified peak ppm values (D, H), while LAVASET evidently assigns much lower importance to those noise features (B, F).

4. ECG DATA

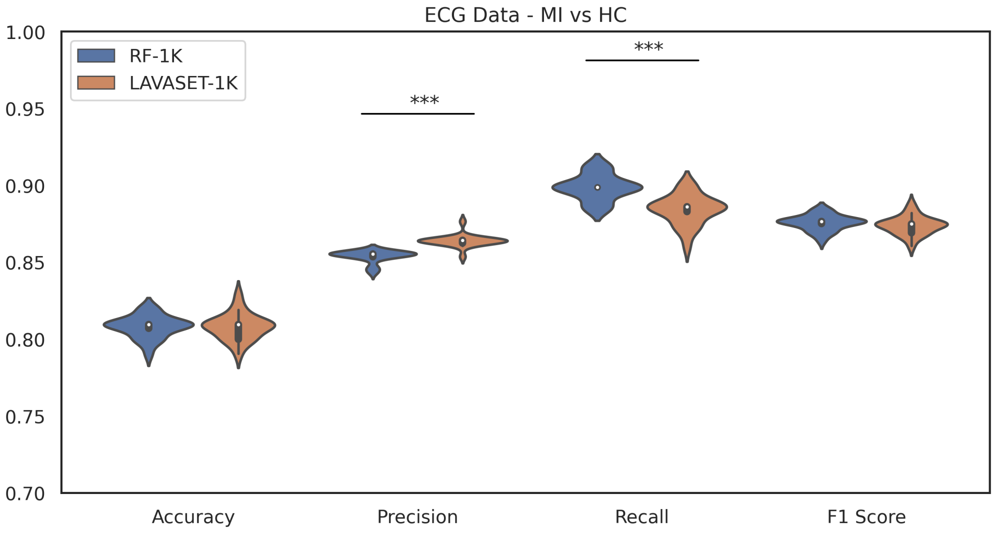

Fig. S8. Performance in the ECG dataset. Models were run for binary classification between Myocardial Infraction (MI) samples and Healthy Controls (HC). Accuracy: LAVASET: 0.81±0.01, RF: 0.81±0.01, Precision: LAVASET: 0.86±0.01, RF: 0.85±0.01, Recall: LAVASET: 0.88±0.01, RF: 0.90±0.01, F1-score: LAVASET: 0.87±0.01, RF: 0.88±0.01.

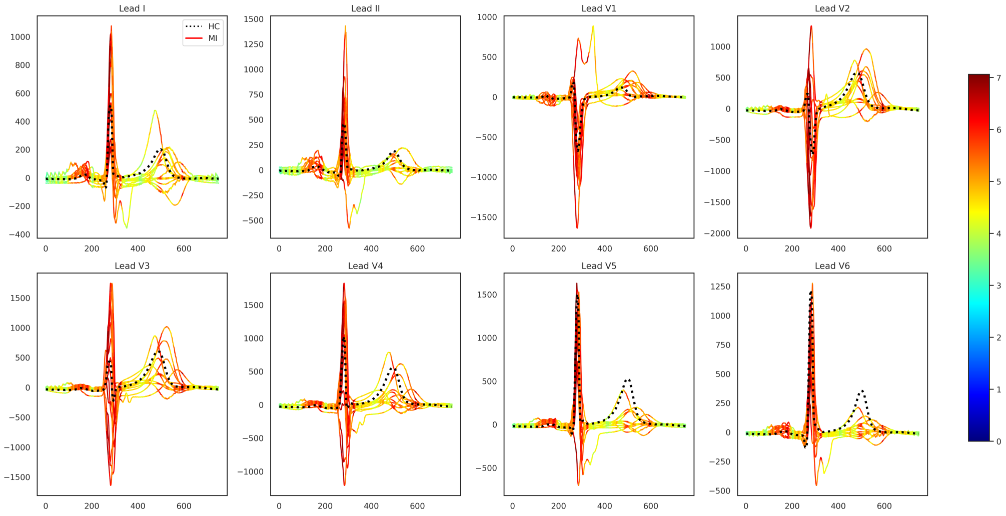

Fig. S9. Feature importance coverage in the ECG dataset. Values are normalised per lead, in order to show the most important areas in the individual leads. Black, dotted lines indicate the (average) Healthy Controls (HCs) and green-yellow-red heatmap-coloured lines show all Myocardial Infarction (MI) cases.
